# “I should” Does Not Mean “I can.” Introducing Efficacy, Normative, and General Compensatory Green Beliefs

**DOI:** 10.1007/s10603-023-09539-6

**Published:** 2023-05-04

**Authors:** M. Penker, S. Seebauer

**Affiliations:** 1 Center for Social Research, University of Graz, Graz, Austria; 2 Life Institute for Climate, Energy Systems and Society, JOANNEUM RESEARCH Forschungsgesellschaft Mbh, Waagner-Biro-Straße 100/9, 8010 Graz, Austria

**Keywords:** Catalysing beliefs, Spillover effect, Mental accounting, Pro-environmental behaviour, Ecological behaviour

## Abstract

Compensatory green beliefs (CGBs) denote beliefs that unsustainable behaviours can be compensated for by performing other sustainable behaviours. We propose to differentiate between efficacy, normative, and general beliefs (ECGBs, NCGBs, GCGBs). ECGBs refer to effectively offsetting previous lapses. NCGBs denote feeling morally obliged to make amends. GCGBs refer to trading off unspecified efforts in overall consumption. Employing survey data from *n* = 502 high school graduates and an *n* = 145 longitudinal subsample, we find a three-factor structure of CGBs. ECGBs, NCGBs, and GCGBs intercorrelate moderately, indicating their status as different constructs. NCGBs are positively associated with pro-environmental values, self-identity, and social norms, whereas GCGBs are negatively associated with these constructs. CGBs, in particular NCGBs, have unique explanatory power for sustainable behaviours. NCGBs show substantial temporal stability over one year. CGBs need not be destructive, as NCGBs may encourage sustainable action. Persuasive messages could be tailored to specific CGBs in specific behavioural domains.

## Compensatory Beliefs in Private Consumption

Compensatory green beliefs (CGBs) denote beliefs that the impact of unsustainable behaviours can be partially or fully compensated for by performing other more sustainable behaviours (Holmgren et al., 2018; Kaklamanou et al., 2015). The concept of compensatory beliefs originates from health research reflecting the premise of different actions contributing to and trading off towards an overall outcome such as “I may eat this piece of cake now because I will exercise this evening” aimed at watching one’s weight (Knäuper et al., 2004). In the domain of environmental protection, CGBs are enacted to protect someone’s environmental credentials or to alleviate negative feelings after giving in to temptation and acting against personal standards for protecting the environment (Byrka & Kaminska, 2015; Hope et al., 2018). Compensatory beliefs are a self-regulatory strategy to reduce cognitive dissonance when failing to meet personal goals (Fanghella & Thøgersen, 2022; Rabiau et al., 2006). Thus, as active self-regulation requires volitional control, compensatory beliefs do not apply to automatic or externally forced behaviours (Radtke et al., 2011).

Reducing the environmental impacts of private consumption is a grand challenge of our time and a central topic of consumer policy. For instance, the pivotal role of consumers in combating climate change is featured prominently in international and national climate policy strategies (European Commission, 2019; Federal Chancellery Austria, 2020). Acts of individual consumption are inherently interwoven and interlinked (Defila & Di Giulio, 2020). Compensatory beliefs could offer entry points for promoting more sustainable consumption patterns by linking specific behaviours. The present paper focuses on compensatory beliefs directed at sustainable behaviours; however, the basic premise of compensatory beliefs and their underlying efficacy, normative, and general dimensions may similarly apply to other fields of consumer behaviour.

Research on CGBs is closely related to the research fields of mental accounting and moral licensing. Mental accounting refers to the balancing of unsustainable actions in one consumption domain against sustainable efforts in other domains (Seebauer, 2018). In mental accounting, consumers balance credits and debits from environmentally relevant actions as they might do with income and expenses in a banking account. Mentally offsetting behaviours is a reason for negative spillover, that is, the performance of one pro-environmental behaviour inhibiting other behaviours (Truelove et al., 2014). In a similar vein, moral licensing refers to the balancing of “good” and “bad” deeds, countering feelings of guilt by emphasizing earlier environmentally friendly actions (Burger et al., 2022; Sorrell et al., 2020). Following the contribution ethic, a person may make an expected contribution to a common goal such as combating climate change and then feel justified in disengaging from further sustainable action (Nash et al., 2017).

In contrast to the perspective of mental accounting and moral licensing on a balance in overall consumption or between consumption domains, however, CGBs are considered to play out between pairs of specific behaviours: a target behaviour, the environmentally harmful lapse, and the compensating behaviour, the pro-environmental behaviour intended to make amends (Bratt, 1999). CGBs are endorsed more strongly if target and compensating behaviour come from similar domains and if the compensating behaviour is easier to perform than the target behaviour (Byrka & Kaminska, 2015; Maki et al., 2019). CGBs need not (and often do not) conform with objective facts and may be liable to compensatory fallacy, that is, wrongly believing that the compensating behaviour is effective for offsetting the actual carbon impact of the target behaviour. For instance, turning off the lights might be rationalized to compensate the carbon impact of holiday air travel. CGBs are positively associated with susceptibility to the negative carbon footprint illusion (MacCutcheon et al., 2020). In most studies, strong CGBs are associated with performing fewer sustainable behaviours (Bratt, 1999; Capstick et al., 2019; Kaklamanou et al., 2015). By contrast, Byrka & Kaminska (2015) observe a positive correlation between CGBs and the General Ecological Behaviour scale (Kaiser, 1998).

### Dimensions of Compensatory Green Beliefs

The present paper argues that these inconsistent effects of CGBs on sustainable behaviour trace back to conceptual ambiguity. We posit that under the umbrella term of CGBs, previous studies measured three different dimensions of CGBs: efficacy, normative, and general CGBs. *Efficacy compensatory green beliefs* (ECGBs) refer to effectively offsetting previous lapses by specific actions (X can compensate for Y; e.g., “Not using a dishwasher can compensate for taking longer showers,” Kaklamanou et al., 2015). *Normative compensatory green beliefs* (NCGBs) denote the perceived moral obligation to make amends by performing specific more sustainable behaviours (If I do X I should do Y; e.g., “If you do not reuse plastic bags, you should use public transportation,” Byrka & Kaminska, 2015). *General compensatory green beliefs* (GCGBs) refer to a holistic perspective on trading off unspecified efforts in overall consumption, similar to mental accounting (e.g., “Doing some things that are positive for the environment means I am allowed to do other things that are less environmentally friendly,” Capstick et al., 2019). Mapping previous empirical studies on CGBs to these three dimensions indicates that Bratt (1999) uses efficacy items; Kaklamanou et al. (2015) predominantly use efficacy items but also some rather normative items (phrased as “if you do X it is okay to do Y”); Capstick et al. (2019) combine efficacy and general items. These three studies observe negative relationships between CGBs and sustainable behaviours. By contrast, Byrka & Kaminska (2015) use normative items and find a positive correlation between CGBs and ecological behaviours. The qualitative study by Hope et al. (2018) highlights the normative underpinning of CGBs because engaging in compensatory efforts implies that “people care about the environment and feel uncomfortable about contributing to environmental problems” (p. 416).

Different results depending on whether efficacy, normative, or general CGBs are measured similarly appear with regard to potential antecedents of CGBs. ECGBs and GCGBs are negatively related to pro-environmental attitudes and worldviews, concerns about climate change, or to a pro-environmental identity (Bratt, 1999; Capstick et al., 2019; Kaklamanou et al., 2015; Nayum & Thøgersen, 2022; Seebauer, 2018). By contrast, as above, NCGBs are positively related to ecological attitudes presumed to underlie the General Ecological Behaviour scale (Byrka & Kaminska, 2015). Few studies addressed other potential antecedents of CGBs: Seebauer (2018) finds a positive relationship between ECGBs and GCGBs and social norms. Kaklamanou et al. (2015) point out that a lack of environmental literacy and knowledge could be a potential reason for the endorsement of CGBs. Environmental self-identity, social norms, pro-environmental values, and environmental knowledge do not just appear as antecedents of CGBs, but are also confirmed drivers of sustainable behaviour (Bamberg & Möser, 2007; Steg & Vlek, 2009; Stern, 2000).

### Aim of the Paper

Thus, the aim of the present paper is to introduce the distinct dimensions of efficacy, normative, and general compensatory green beliefs as an approach to resolve conceptual ambiguity in previous research. In order to establish these three dimensions, we explore the validity and effects of respective CGB measures, drawing on survey data from 502 Austrian high school graduates and employing confirmatory factor analysis and structural equation modelling. We establish construct validity by confirming the efficacy, normative, and general dimensions as separate factors. We discuss their discriminant validity by showing that CGBs do not overlap with their antecedents’ environmental self-identity, social norms, pro-environmental values, and environmental knowledge. We discuss their incremental validity by showing that CGBs have additional explanatory power for sustainable behaviours beyond these antecedents. A longitudinal subsample of 145 students points to high temporal stability of NCGBs and cautions against interpreting cross-sectional correlations as causal effects.

We argue that compensatory green beliefs need not be destructive; on the contrary, normative CGBs may encourage rather than undermine individual sustainable action. We conclude by pointing out directions how compensatory beliefs, which are still a niche topic in research on sustainable consumption, could inform larger research on the discrepancy between the subjective accounting and licensing of individual efforts and the objective environmental and carbon impact these efforts actually have. The present study should be considered exploratory as it followed up on ambiguity detected in previous CGB research but did not state and preregister a priori hypotheses.

## Method

### Data

Standardized self-completion questionnaires were distributed from February to May 2020 to students in their final high school year (12th or 13th year of formal education), aged 17 to 21 years. The survey was implemented in 24 vocational or general secondary schools in urban and rural locations in the Austrian provinces of Styria and Tyrol. Schools were approached via the researchers’ personal network and upon recommendation by provincial school authorities (snowballing method). Students completed an online questionnaire in the classroom during school hours, using the school’s computers or their own electronic devices. A researcher was present on-site for oversight and clarification. Because of school closures in the Covid-19 national lockdown starting in mid-March 2020, however, data collection had to shift to an entirely online survey: Teachers distributed an email invitation to the online survey and up to two reminders to their respective students who completed the questionnaire as a homeschooling exercise. Out of *n* = 502 valid responses in total, 63.5% were collected in the classroom setting and 36.5% were completed at home.

One year later, in March to April 2021, those 355 students who had agreed to participate in a follow-up survey were approached again. These students received an email invitation to an online questionnaire and three reminder emails, plus a reminder text message if they had provided a mobile phone number. Students were offered participation in a lottery of gift vouchers (8 × 50 Euro) as an incentive to take part in the survey. Contact data of 19 students was invalid. In total, 43% of the respondents who had provided valid contact data participated in the follow-up survey, yielding a longitudinal sample of *n* = 145 cases. During the year between the first survey wave in 2020 (t1) and the second survey wave in 2021 (t2), the respondents experienced a formative biographical phase wherein several life events coincided: high school graduation (98.6% experienced this life event), moving out of the parental home (35.9%), doing military/civil service (29.6%; compulsory for able-bodied male citizens), taking up gainful employment (51.0%), and/or entering academic education (57.2%). Respondents experienced on average 2.73 life events (SD = 0.85).

Table 6 in the Appendix gives the distribution by gender, age, and education of parents in the samples at t1 vs. t2 and completed in the classroom vs. at home. Socio-demographics in the first survey wave subsamples completed in the classroom vs. at home conformed fairly well; however, participation in the second survey wave seemed biased by higher education of parents.

The sample stemmed from a larger project on climate attitudes and behaviours among young adults in Austria. Analysing high school graduates rather than the general population as done in earlier CGB studies might bias the results. However, Mayerl & Best (2019) point out that the evidence on the relationship between age and environmental attitudes is mixed, with some authors finding a positive relationship, others a negative relationship, and others no relationship at all. Our own analysis of the 2020 ISSP Environment Module for Austria (ISSP Research Group, 2022) shows no substantial association between age and environmental attitudes (in terms of environmental concern and willingness to sacrifice). Students can be expected to have agency and control in the behaviours addressed in our CGB measures. We therefore presume that our results may generalize to the general population; however, this assumption remains to be confirmed in future studies.

### Measures

All constructs were measured using multiple items, to correct for measurement error of single items. For exact wordings and descriptive statistics of all items, please refer to Table 1 for NCGBs and ECGBs and to Table 7 in the Appendix for all other items. Responses were measured on a rating scale from fully agree (5) to fully disagree (1), except for sustainable behaviours which were measured from always (5) to never (1). Items were presented in mixed order in the questionnaire, so that it was not transparent to the respondents which item was assigned to which construct; however, the NCGB and ECGB items were grouped in respective blocks. All items were originally presented in German and introduced as part of a survey on everyday climate-relevant behaviours and opinions on climate protection. Items were pre-tested with seven students before t1 and with fifteen students before t2 field work to ensure unambiguous language and comprehensibility. Neither in the pre-tests nor during in-classroom data collection were any difficulties in comprehension observed.

**Table 1:** Descriptive statistics

NCGB	Mean	SD	ECGB	Mean	SD	Correlation NCGB-ECGB
If I take a full bath once a week, I should not shower too long on the remaining days. (NCGB1)	3.88	1.28	I can reduce the energy consumption of a weekly full bath by taking a short shower on the remaining days. (ECGB1)	3.62	1.17	0.21 [0.12, 0.29]
When I fly abroad, I should only use public transport at home. (NCGB2)	3.08	1.30	I can reduce the climate impact of my flights abroad by using only public transport at home. (ECGB2)	3.18	1.28	0.36 [0.27, 0.43]
If I buy imported products at the supermarket, I should get there on foot or by bicycle. (NCGB3)	2.93	1.33	I can reduce the climate impact of imported products by walking or cycling to the supermarket. (ECGB3)	3.04	1.26	0.27 [0.18, 0.35]
I should compensate for the purchase of new electrical devices by always disconnecting these devices from the mains when not using them. (NCGB4)	3.55	1.27	I can compensate for the purchase of new electrical products by always disconnecting these devices from the mains when not using them. (ECGB4)	3.22	1.29	0.43 [0.35, 0.50]
If I travel by air, I should pay a CO_2_ compensation. (NCGB5)	3.24	1.38	I can reduce the climate impact of air travel by paying a CO_2_ compensation. (ECGB5)	2.62	1.38	0.32 [0.23, 0.40]

*SD*, standard deviation. *N* = 476–482, one-wave cross-sectional data. All Pearson’s correlations are significant at *p* < 0.001. 95% confidence interval in square brackets. Items NCGB1 and ECGB1 refer to energy saving by curbing hot water use, not overall water saving

#### Normative and Efficacy Compensatory Green Beliefs

Five items were used, each pairing a target behaviour with a compensating behaviour. In order to capture compensatory fallacy, the paired behaviours were deliberately selected for it to be fairly unlikely within most living situations that the compensating behaviour would actually offset the target behaviour in terms of real-world carbon impact. Items were matched between the NCGB and ECGB construct, phrasing the same pair of behaviours as normative (“I should”) and as efficacy (“I can”). All items were phrased in the first person to make them more personally relevant (Kaklamanou et al., 2015). ECGB items were anchored to climate impact or energy consumption as a tangible physical reference (Hope et al., 2018). All items referred to behaviours that are under the students’ control even while still living in the parental home. Behaviours were selected to cover diverse consumption domains. In order to limit respondent burden, only normative CGBs were included at t2 because NCGBs showed the strongest effects in incremental validity (see the “Incremental Validity” section). The three normative items with the highest factor loadings at t1 were repeated at t2 (NCGB2, NCGB3, NCGB4).

#### General Compensatory Green Beliefs

General Compensatory Green Beliefs were measured by two items, indicating the view that a few climate-friendly activities make it permissible to keep up the current lifestyle (Capstick et al., 2019). GCGBs were not measured at t2.

#### Environmental Knowledge

Respondents were asked eight quiz questions about effective carbon saving, each quiz question featuring three multiple-choice options with one correct answer (Frick et al., 2004). The quiz questions were aggregated formatively to a manifest sum score of correct answers, ranging from 0 to 8, with higher scores indicating better knowledge. Environmental knowledge was not measured at t2.

#### Environmental Self-Identity

Self-identity refers to the picture people hold of themselves. Three items expressed a sense of being a climate-friendly person and normative feelings of responsibility and obligation towards climate protection (Steinhorst et al., 2015; van der Werff et al., 2014).

#### Social Norms

Social norms refer to the perceived expectations of important others. Four items measured descriptive and injunctive social norms for climate protection conveyed by people who are important for or close to the respondent (Klöckner et al., 2013; Wolf & Seebauer, 2014).

#### Pro-environmental Values

Pro-environmental values address general views on the relationship between humans and nature. Three items from the New Environmental Paradigm scale (Amburgey & Thoman, 2012; Dunlap, 2008) were included; due to space restrictions, only the two items with the highest factor loadings at t1 were repeated at t2. For analysis, the value items were reverse-coded so that higher numerics refer to stronger pro-environmental values.

#### Sustainable Behaviours

Self-reported frequency of everyday activities was measured in the domains food consumption (four items), indulging (three items), waste separation (two items), repair and reuse (three items), and energy use (four items) (Seebauer et al., 2017). Activities were selected to cover behaviours of substantial carbon impact as well as pro-environmental meaning and to ensure that students have agency in these behaviours while still living in the parental home. Behaviours were aggregated to five domains because Capstick et al. (2019) point to differential effects of CGBs by behavioural domain. For indulging behaviours, higher numerics refer to less sustainable behaviour. For comparison, all behavioural items were reverse-coded if necessary and then averaged to a general behaviour formative index. The resulting score was subsequently entered as observed variable into the structural equation models. Note that only the energy use items included behaviours that are featured as compensating or target behaviour in the NCGB/ECGB items (save hot water, disconnect electrical devices instead of standby mode).

### Analytical Approach

The analysis proceeds in five steps (these steps correspond to the subsections under the “Results” section): (1) We present descriptive statistics on NCGB and ECGB items to illustrate how strongly compensatory beliefs are endorsed in our study sample. Next, we employ confirmatory factor analysis to (2) ascertain the three-dimensional structure of normative, efficacy, and general CGBs we posit based on our review of previous CGB research (construct validity) and to (3) differentiate CGBs from the related but conceptually distinct constructs environmental knowledge, self-identity, social norms, and values (discriminant validity). Then, (4) structural equation modelling assesses whether CGBs have additional, unique explanatory power for sustainable behaviours above and beyond environmental knowledge, self-identity, social norms, and values (incremental validity). In the final step (5), we calculate cross-lagged autoregressive models, each model combining NCGBs with another normative or behavioural latent variable, in order to analyse temporal stability and the direction of causal effects. In step (5), we report the difference in the χ^2^ model fit statistic between a restricted model that assumes equality of both cross-lagged path coefficients and an unrestricted model, where the two coefficients are estimated freely; if the restricted model fits significantly worse, this indicates a direction of influence under the presumption that the time between cause (here: t1) and effect (here: t2) ascertains causal direction (Finkel, 1995). However, autoregressive effects only provide information about the relative stability of rank ordering over time and do not allow to infer changes at the aggregate level (Newsom, 2015). We therefore compare NCGB means at t1 and t2 in a *t*-test for paired samples.

Steps (1) to (4) use the one-wave cross-sectional data from *n* = 502 cases at t1 only. Steps (1) to (3) use a reduced sample of 482 cases because 20 cases did not give any responses on the CGB variables. Step (5) uses the two-wave longitudinal data from *n* = 145 cases at t1 and t2. The small longitudinal sample may, however, only yield tentative results: With 12–14 observed variables and 48–55 free parameters to be estimated in each cross-lagged autoregressive model, the sample size complies with the rule of thumb of at least 10 cases per observed variable but falls short of the recommendation of at least 5 cases per free parameter (Bentler & Chou, 1987; Dilalla, 2000) and is therefore presumably underpowered.

The confirmatory factor analysis used in steps (2) and (3) includes error covariances between paired NCGB-ECGB items to reflect a method effect of similar phrasing (Brown, 2015). The step (5) cross-lagged autoregressive models include error covariances between the same items at t1 and t2 in order to account for temporal stability of measurement error (Finkel, 1995). The quality of the assignment of items to the environmental self-identity, social norms, pro-environmental values, and sustainable behaviours latent variables is reflected in the general model fit; therefore, results from separate confirmatory factor analyses are omitted. Factor loadings are satisfactory throughout (mostly > 0.50; see Table 7 in the Appendix). The step (4) models include covariances between all exogenous latent variables.

All structural equation models are calculated with raw data, using full information maximum likelihood (FIML) estimation with robust standard errors, and robust/scaled test statistics and fit indices to account for missing values and non-normality implemented in the lavaan R package (Rosseel, 2021). Model fit indices indicate how well the observed data are represented by the model structure. Model fit is considered good with a ratio of χ^2^ to degrees of freedom (df) lower than 5, a root mean square error of approximation (RMSEA) lower than 0.08, a standardized root mean square residual (SRMR) lower than 0.05, and a comparative fit index (CFI) larger than 0.95 (Byrne, 2010). All results are tested against a *p* < 0.05 significance level. Point estimates of coefficients are presented with 95% normal theory confidence intervals.

In structural equation modelling, power depends not just on the size of the targeted effect and the size of the sample, but also on the structure and parameters of the entire model (Wang & Rhemtulla, 2021). We estimate power of the steps (3) and (4) using the pwrSEM application by Wang & Rhemtulla (2021) under specific parameter assumptions (see Table 8 in the Appendix). The sample is large enough to detect correlations between latent variables of 0.30 and standardized CGB path coefficients of 0.20; however, for GCGB, the study is slightly underpowered since this construct is measured with just two items.

## Results

### Endorsement

Mean scores show endorsement of NCGBs and ECGBs in the mid-range of the five-step response scale (Table 1). Respondents slightly favour compensatory beliefs referring to household energy consumption (hot water use, disconnecting electrical devices) over beliefs referring to transport. Similar to previous CGB studies, notwithstanding the different survey populations these studies investigated, NCGB endorsement tends to be higher than ECGB endorsement (Bratt, 1999; Byrka & Kaminska, 2015; Kaklamanou et al., 2015). Variance is fairly high in all NCGB and ECGB items (SD > 1.2), pointing to heterogeneity in the degree to which high school students hold compensatory beliefs that may be explained by other psychological constructs in the following sections. Correlations from *r* = 0.21 to *r* = 0.43 suggest that normative and efficacy beliefs are related but conceptually different.

### Construct Validity

Confirmatory factor analysis corroborates the three-dimensional structure of normative, efficacy, and general CGBs as interrelated but separate factors, as indicated by good model fit (Table 2). In all three factors, the average variance extracted fulfils the Fornell-Larcker criterion of being higher than the squared correlations with other factors (Fornell & Larcker, 1981). Factor loadings are satisfactory in NCGBs and ECGBs; however, the weaker loadings in GCGBs suggest a higher measurement error when measuring CGBs as a holistic view on balancing climate actions than when specifying the involved compensating and target behaviours.

**Table 2:** Confirmatory factor analysis of normative, efficacy, and general CGBs

	Coefficient	95% CI	AVE	CR
Normative beliefs			0.46	0.80
NCGB1	0.55***	[0.48, 0.62]		
NCGB2	0.81***	[0.76, 0.86]		
NCGB3	0.80***	[0.75, 0.84]		
NCGB4	0.65***	[0.59, 0.72]		
NCGB5	0.53***	[0.44, 0.61]		
Efficacy beliefs			0.49	0.82
ECGB1	0.70***	[0.63, 0.76]		
ECGB2	0.78***	[0.72, 0.84]		
ECGB3	0.78***	[0.72, 0.84]		
ECGB4	0.68***	[0.61, 0.75]		
ECGB5	0.55***	[0.47, 0.62]		
General beliefs			0.35	0.28
GCGB1	0.79***	[0.43, 1.15]		
GCGB2	0.30**	[0.11, 0.49]		
Latent variable correlations				
NCGB-ECGB	0.39***	[0.27, 0.50]		
NCGB-GCGB	− 0.26**	[− 0.43, − 0.10]		
ECGB-GCGB	0.15	[− 0.07, 0.37]		

Table 2 gives standardized factor loadings and latent variable correlations. *N* = 482, one-wave cross-sectional data. *** *p* < 0.001; ** *p* < 0.01. 95% confidence interval in square brackets. χ.^2^ = 333.3, df = 205; RMSEA = 0.037 [90% CI: 0.030; 0.044]; SRMR = 0.049; CFI = 0.963. *AVE*, average variance extracted. *CR*, composite reliability. Table 2 refers to the same confirmatory factor analysis as in Table 3

The correlation of *r* = 0.39 between NCGBs and ECGBs mirrors the single-item correlations in Table 1. The negative correlation of *r* =  − 0.26 between NCGBs and GCGBs suggests that feeling morally obliged to compensate for specific lapses runs counter to a general sense of entitlement and excuse of having already contributed enough to climate protection. Although not statistically significant, the positive correlation of *r* = 0.15 between ECGBs and GCGBs indicates that (erroneously) believing in the actual compensatory effect of everyday activities may coincide with a general view that current personal endeavours suffice for doing one’s part in climate protection.

A principal component analysis, conducted as a robustness check, supports normative, efficacy, and general CGBs as three distinct dimensions. ECGB, NCGB, and GCGB items show clear loadings on the respective factors (Table 9 in the Appendix). As a second robustness check, a confirmatory factor analysis measuring NCGB and ECGB as multi-item constructs as in Table 2, but measuring GCGB as a single-item construct using only the more reliable GCGB1 item yields similar results (Table 10 in the Appendix).

### Discriminant Validity

The confirmatory factor analysis also includes correlations between CGBs and environmental knowledge, self-identity, social norms, and values as constructs related to CGBs. As shown in Table 3, CGBs are moderately correlated but do not overlap with these constructs. In line with previous research (see the “Compensatory Beliefs in Private Consumption” section), NCGBs correlate positively (*r* = 0.32 to *r* = 0.49) and GCGBs correlate negatively (*r* =  − 0.35 to *r* =  − 0.39) with pro-environmental norms and values. ECGBs, however, contrary to the negative relationship observed in Bratt (1999), Kaklamanou et al. (2015), and Seebauer (2018), are practically unrelated to pro-environmental norms and values; the *r* = 0.16 correlation of ECGB with self-identity presumably arises from a linear combination of the ECGB-NCGB and NCGB-self-identity correlations.

**Table 3:** Confirmatory factor analysis of normative, efficacy, and general CGBs with environmental knowledge, environmental self-identity, social norms, and pro-environmental values

	NCGB	ECGB	GCGB
Environmental knowledge	0.03 [− 0.07, 0.12]	− 0.12* [− 0.22, − 0.02]	− 0.16* [− 0.31, − 0.01]
Environmental self-identity	0.49*** [0.40, 0.59]	0.16** [0.03, 0.28]	− 0.39*** [− 0.55, − 0.24]
Social norms	0.32*** [0.21, 0.42]	0.11 [− 0.01, 0.23]	− 0.35*** [− 0.48, − 0.22]
Pro-environmental values	0.35*** [0.22, 0.49]	0.09 [− 0.05, 0.24]	− 0.38** [− 0.63, − 0.13]

Table 3 gives latent variable correlations. *N* = 482, one-wave cross-sectional data. ***p < 0.001; **p < 0.01; *p < 0.05. 95% confidence interval in square brackets. χ.^2^ = 333.3, df = 205; RMSEA = 0.037 [90% CI: 0.030; 0.044]; SRMR = 0.049; CFI = 0.963. Table 3 refers to the same confirmatory factor analysis as in Table 2

Both in ECGBs and GCGBs, weaker compensatory beliefs are associated with a higher level of environmental knowledge (*r* =  − 0.12 and *r* =  − 0.16). Being literate about the actual energy consumption and carbon emissions of everyday activities seems to correct an overly optimistic mindset that minor adjustments in daily consumption suffice to balance the personal carbon footprint. A robustness check of the Table 3 confirmatory factor analysis using only the GCGB1 item yields similar results (Table 11 in the Appendix).

### Incremental Validity

Next, structural equation modelling shows that CGBs have additional, unique explanatory power for explaining sustainable behaviours in various domains. In most behavioural domains, CGBs increase explained variance *R*^2^ by a third when controlling for the influence of environmental knowledge, self-identity, social norms, and values (see Table 4). NCGBs stand out, as they show significant positive effects on sustainable behaviour in four out of five domains (β = 0.22 to β = 0.36). NCGBs show the strongest effect on energy use (β = 0.36), most likely because the NCGB1 and NCGB4 items directly refer to household energy consumption. The unique effect of NCGBs additional to the influence of self-identity and social norms underscores that NCGBs imply a perceived moral obligation beyond common normative expectations. To some extent, the explanatory advantage of NCGBs over self-identity could be attributed to higher correspondence with the explained behavioural domains: NCGB items refer to specific behaviours, but self-identity items refer to an unspecific sense of being a climate-friendly person. However, since NCGBs are measured as a latent variable comprising behaviours from different domains, an eventual bias is presumably rather small.

**Table 4:** Structural equation modelling explaining sustainable behaviours from normative, efficacy, and general CGBs, and other constructs

	Food consumption	Indulging	Waste separation	Repair and reuse	Energy use	General behaviour
Environmental knowledge	− 0.01 [− 0.10, 0.08]	− 0.16** [− 0.27, − 0.05]	− 0.05 [− 0.18, 0.08]	0.02 [− 0.09, 0.14]	− 0.13 [− 0.24, − 0.02]	− 0.11** [− 0.18,. − 03]
Environmental self-identity	0.51*** [0.28, 0.74]	0.02 [− 0.25, 0.29]	0.57** [0.19, 0.95]	0.14 [− 0.12, 0.40]	0.50*** [0.21, 0.79]	0.48*** [0.27, 0.68]
Social norms	0.29*** [0.14, 0.43]	0.04 [− 0.13, 0.21]	− 0.19 [− 0.43, 0.04]	0.08 [− 0.10, 0.26]	− 0.06 [− 0.24, 0.11]	0.05 [− 0.08, 0.18]
Pro-environmental values	0.04 [− 0.17, 0.26]	− 0.08 [− 0.35, 0.18]	− 0.04 [− 0.34, 0.26]	− 0.02 [− 0.25, 0.22]	− 0.16 [− 0.44, 0.12]	− 0.08 [− 0.26, 0.10]
NCGB	0.14 [− 0.01, 0.28]	− 0.27** [− 0.47, − 0.07]	0.34** [0.09, 0.60]	0.22* [0.04, 0.40]	0.36*** [0.17, 0.54]	0.21*** [0.09, 0.34]
ECGB	− 0.06 [− 0.19, 0.07]	0.23** [0.09, 0.38]	− 0.09 [− 0.27, 0.08]	− 0.06 [− 0.23, 0.11]	0.03 [− 0.13, 0.18]	0.00 [− 0.10, 0.12]
GCGB	− 0.04 [− 0.22, 0.15]	0.05 [− 0.13, 0.24]	0.13 [− 0.09, 0.35]	− 0.18 [− 0.39, 0.03]	− 0.01 [− 0.21, 0.18]	− 0.03 [− 0.16, 0.10]
R^2^ (R^2^ without CGB)	0.69 (0.67)	0.15 (0.04)	0.39 (0.27)	0.20 (0.12)	0.43 (0.32)	0.38 (0.35)
χ^2^ (df)	502.1 (292)	448.9 (267)	391.3 (243)	402.4 (267)	490.2 (292)	373.6 (221)
RMSEA [90% CI]	0.040 [0.033, 0.045]	0.038 [0.032, 0.045]	0.036 [0.029, 0.043]	0.033 [0.026, 0.040]	0.039 [0.032, 0.045]	0.039 [0.032, 0.046]
SRMR	0.051	0.053	0.047	0.047	0.049	0.049
CFI	0.949	0.952	0.959	0.963	0.947	0.959

Table 4 gives standardized path coefficients. *N* = 502, one-wave cross-sectional data. ***p < 0.001; **p < 0.01; *p < 0.05. 95% confidence interval in square brackets. Specific behaviours are measured as reflective latent variables, whereas general behaviour is measured as a formative observed variable

ECGBs seem to counteract pro-environmental action by incurring carbon-intensive indulging behaviours (β = 0.23). That ECGBs are particularly related with the indulging domain could indicate the compensatory premise of allowing oneself indulgence as unsustainable lapses may be easily corrected by subsequent minor adjustments. The positive effect of NCGBs and the negative effect of ECGBs on sustainable behaviour correspond with previous studies (see the “Compensatory Beliefs in Private Consumption” section). GCGBs are not significantly associated with any domain of sustainable behaviour, presumably because general CGBs do not refer to specific behaviours as normative and efficacy CGBs do. These effects hold in a robustness check using the GCGB1 item as a single-item measure of the GCGB construct (Table 12 in the Appendix), with the only exception of a statistically significant yet smaller effect of GCGB on repair and reuse behaviours. An overall perspective on trading off efforts at acting in a sustainable manner might primarily affect behaviours that imply making do with current possessions.

Apart from the CGB effects, the effects of the other predictors reflect pertinent research: Environmental knowledge has a marginal effect (β <|.16|; Frick et al., 2004). Self-identity is a significant predictor in the low-cost domains of food consumption, waste separation, and energy use behaviours (β = 0.50 to β = 0.57; Diekmann & Preisendörfer, 1998; van der Werff et al., 2014) but not the other domains. Food consumption might be enacted for its public visibility and for interpersonal distinction and is consequently related to social norms (β = 0.29; Welsch & Kühling, 2009). Overarching pro-environmental values are hardly translated into behaviour-specific action (β <|.16|; Bamberg & Möser, 2007). Possibly, the dominant effects of self-identity (β = 0.51) and social norms (β = 0.29) on food consumption suppress NCGBs, incurring a comparatively weak and non-significant effect of NCGBs (β = 0.14).

The model for explaining general behaviour as the average of all analysed sustainable behaviours mostly mirrors the domain-specific results: Self-identity and NCGBs, which have significant effects across most behavioural domains, retain their effects with regard to general behaviour. The effect of environmental knowledge seems to carry over from the indulging domain. Social norms and ECGBs, which influence only selected behavioural domains, however, seem to be levelled out when focusing on general behaviour.

### Temporal Stability

In the final analytical step, the two-wave longitudinal subsample illustrates substantial temporal stability of NCGBs but cannot determine causal directions between NCGBs and other constructs (Table 5). NCGBs as well as self-identity, social norms, and sustainable behaviours show considerable autoregressive effects, underscoring that NCGBs remain mostly unchanged (β = 0.47 to β = 0.54) and norms and behaviours are almost constant over one year from t1 to t2 (β = 0.75 to β = 0.98; Table 5). As a reference, previous studies on public transport use report stabilities of around β = 0.40 to β = 0.50 in attitudes and social norms and around β = 0.50 to β = 0.80 in behaviours over timeframes of four months to one year (Bamberg et al., 2003; Klöckner et al., 2003; Thøgersen, 2006). Judging from the path coefficient effect sizes, NCGBs seem as stable as other environmental attitudes. The means of an index averaging all NCGB items do not differ between t1 and t2 (*M*_t1_ = 3.34; *M*_t2_ = 3.34; *t*(144) = 0.09, *p* = 0.93). It comes as no surprise that environmental beliefs and behaviours persist over time also in the present study (Stern, 2000; Verplanken & Orbell, 2003). Still, the observed high stabilities are still remarkable because of the substantial biographical change undergone by the study population of high school graduates. Experiencing multiple life events during the year from t1 to t2 could be expected to encourage revision and adjustment of ingrained practices (Beige & Axhausen, 2012; Schäfer et al., 2012). That NCGBs persist under changing circumstances speaks to their status as an enduring mindset.

**Table 5:** Cross-lagged autoregressive models on normative CGBs and other constructs

		Constructs					
		Self-identity	Social norms	Food consumption	Repair and reuse	Energy use	General behaviour
Autoregressive effect	Construct t1 → construct t2	0.86*** [0.76, 0.96]	0.80*** [ 0.68, 0.92]	0.98*** [0.84, 1.11]	0.88*** [0.68, 1.07]	0.97*** [0.74, 1.19]	0.75*** [0.66, 0.84]
	NCGB t1 → NCGB t2	0.52*** [0.32, 0.73]	0.50*** [0.31, 0.70]	0.47*** [0.25, 0.70]	0.54*** [0.34, 0.74]	0.50*** [0.28, 0.73]	0.52*** [0.32, 0.72]
Correlation	Construct t1 – NCGB t1	0.36*** [0.17, 0.55]	0.26** [0.05, 0.47]	0.40** [0.19, 0.61]	0.22 [− 0.08, 0.53]	0.28* [0.02, 0.54]	0.33*** [0.16, 0.50]
	Construct t2 – NCGB t2	0.28 [− 0.06, 0.53]	− 0.07 [− 0.35, 0.20]	0.34 [− 0.14, 0.82]	0.10 [− 0.51, 0.74]	− 0.06 [− 0.85, 0.73]	0.21* [0.01, 0.41]
Cross-lagged effect	Construct t1 → NCGB t2	0.00 [− 0.22, 0.23]	0.10 [− 0.09,0.30]	0.13 [− 0.13, 0.39]	− 0.04 [− 0.30, 0.22]	0.07 [− 0.27, 0.43]	− 0.01 [− 0.20, 0.18]
	NCGB t1 → construct t2	− 0.02 [− 0.16, 0.13]	− 0.02 [− 0.17, 0.14]	− 0.14 [− 0.34, 0.07]	0.01 [− 0.24, 0.26]	− 0.07 [− 0.33, 0.14]	− 0.00 [− 0.13, 0.11]
	*R*^2^ in construct t2	0.73	0.64	0.86	0.77	0.89	0.56
	*R*^2^ in NCGB t2	0.27	0.29	0.29	0.28	0.28	0.27
	χ^2^ (df)	53.7 (44)	81.0 (66)	84.8 (66)	48.9 (44)	91.2 (66)	26.8 (14)
	Diff in χ^2^	0.01	0.91	1.65	0.11	0.65	0.01
	RMSEA [90% CI]	0.037 [0.000, 0.071]	0.037 [0.000, 0.067]	0.042 [0.000, 0.069]	0.019 [0.000, 0.060]	0.050 [0.013, 0.076]	0.076 [0.016, 0.124]
	SRMR	0.062	0.047	0.059	0.050	0.071	0.55
	CFI	0.99	0.98	0.97	0.99	0.95	0.97

Table 5 gives standardized path coefficients and latent variable correlations. Correlations at t2 refer to correlations between construct residuals. *N* = 145, two-wave longitudinal data. ***p < 0.001; **p < 0.01; *p < 0.05. 95% confidence interval in square brackets. Diff in χ^2^ = model comparison for equal cross-lagged paths, df = 1. The models including pro-environmental values, indulging, and waste separation do not converge because of negative error variances in the respective constructs. Specific behaviours are measured as reflective latent variables, whereas general behaviour is measured as a formative observed variable

The correlations of NCGBs with self-identity and social norms at t1 (*r* = 0.36 and *r* = 0.26, respectively) reflect the results on discriminant validity in Table 3. The correlations between NCGBs and other constructs (between exogenous constructs at t1, and between construct residuals at t2) point to a certain influence of unmeasured joint background variables (Finkel, 1995). Presumably, correlations at t2 are smaller than at t1 because the residual correlations are corrected by the shared variance from cross-lagged effects, even if these cross-lagged effects are small.

The high temporal stability leaves little remaining variance at t2 to be explained. Consequently, the cross-lagged path coefficients are weak and difference in χ^2^ from a model assuming equal cross-lagged paths is low. The models do not consistently show higher cross-lagged effects from the more stable constructs to the less stable NCGBs than vice versa, as might be expected. Thus, causal direction between NCGBs on the one hand and self-identity, social norms, or sustainable behaviours, on the other hand, cannot be determined. This cautions against interpreting the incremental validity results in Table 4 as causal effects, since the results from the cross-sectional data are not replicated in the small and most likely underpowered longitudinal sample. Judging by the cross-lagged effects of similar size but opposing signs between NCGBs on the one hand and food consumption and energy use on the other hand, we may speculate that NCGBs and sustainable behaviour are balanced against each other over time, mitigating behavioural transgressions by increased normative expectations (in a similar way to the moral licensing logic, see the “Compensatory Beliefs in Private Consumption” Sect. 1). These potential trade-off effects are not, however, supported by statistical significance.

The respondents in the longitudinal sample self-selected for participation in the second survey wave and therefore might be biased towards sustainable attitudes and behaviours. Mean levels at t1 are marginally higher among the 145 s-wave participants than among the remaining 357 respondents of the cross-sectional sample (+ 0.2 to + 0.4 steps on the five-step rating scale in food consumption, repair and reuse, and energy use behaviours; + 0.5 scale steps in self-identity, social norms; + 0.3 to + 0.4 scale steps in NCGBs). Even though means are higher among second-wave participants than among the other respondents, the data do not indicate a ceiling effect. If the second-wave participants’ responses crowd in the upper range of the rating scale, this would limit variance and could lead to overestimated stability coefficients. However, mean levels of second-wave participants at t1 are well below four on the five-step rating scale (see Table 7 in the Appendix; apart from the energy use behaviour of turning off the lights, which is strongly endorsed by all respondents).

## Discussion

In order to address conceptual ambiguity in previous research on compensatory green beliefs, we introduce a three-dimensional perspective. In our study sample, normative, efficacy, and general compensatory green beliefs can be differentiated among themselves (construct validity) and from the constructs environmental knowledge, self-identity, social norms, and values (discriminant validity). CGBs, in particular NCGBs, have unique explanatory power for sustainable behaviours, above and beyond the norms, values, and knowledge constructs (incremental validity). NCGBs hardly change over time, which supports their status as underlying, persistent beliefs (temporal stability).

However, these findings should be taken only as a starting point for subsequent research on the role of compensatory beliefs in consumer choices. The triad of normative, efficacy, and general compensatory beliefs needs to be replicated for additional domains of sustainable behaviour and for consumer behaviours unrelated to sustainability or climate protection, for other countries than Austria, for other populations than high school graduates, and with larger random samples than a convenience sample as in the present study. Surveying the incidence of CGBs in the general population would yield more heterogeneity in socio-demographics and could indicate which population segments hold more or fewer CGBs. Longitudinal or experimental studies could analyse the role of CGBs before and after lapses by observing how compensatory beliefs are implemented as manifest compensatory actions, such as paying extra for carbon certificates when buying a plane ticket, or engaging in less environmental behaviours after acquiring an electric vehicle (Nayum & Thøgersen, 2022). Future studies should include other related concepts such as general self-efficacy or control beliefs, since our focus on normative concepts (self-identity, social norms, values) mainly accounts for the validity of normative CGBs. In any case, to facilitate comparison and integration of diverging evidence, we suggest for future studies that it be explicitly stated which specific CGB dimensions they are investigating and that items be phrased accordingly.

## Conclusions

### Implications for Promoting Sustainable Behaviour

With regard to promoting sustainable behaviour in persuasive interventions, the results indicate both a constructive and a destructive facet of compensatory green beliefs. Normative CGBs are positively associated with sustainable behaviour; this constructive facet emerges most strongly in waste separation and energy use. General CGBs and, in a less pronounced way, efficacy CGBs are negatively associated with sustainable behaviour; this destructive facet appears most strongly in indulging and repair and reuse behaviours. People who reflect on their unsustainable behaviour are more likely to be persuaded to change their behaviour (Hope et al., 2018). Persuasive messages could be tailored to normative or efficacy CGBs depending on the specific behavioural domains targeted. Compensatory beliefs are discussed as a spillover mechanism (Nash et al., 2017); thus, making NCGBs salient may inhibit negative spillover. However, taking moral actions to compensate for previous immoral actions, as NCGBs suggest, may be enacted less in the environmental than in other domains (Fanghella & Thøgersen, 2022). Judging from the discriminant validity correlations (Table 3), NCGBs could be advanced by appealing to self-identity, social norms, and pro-environmental values. ECGBs and GCGBs reflect how compensatory beliefs function as a mechanism for coping or even self-deception—if someone wants to relieve their bad conscience, they have to convince themselves that their compensatory actions actually suffice to correct their lapse, even if this belief is inconsistent with common sense or carbon footprint calculations. Environmental knowledge is negatively associated with ECGBs and GCGBs (Table 3), presumably because knowing the facts about real-world carbon impacts makes it hard to uphold erroneous beliefs. Thus, educating individuals on the actual carbon impact of their actions, for instance by product labelling, could help in debunking compensatory fallacy and could support realistic mental accounting of personal contributions to climate protection.

CGBs are characterized by pairs of specific behaviours: an environmentally harmful target behaviour is mentally linked to an environmentally friendly compensating behaviour. Identifying pairs of behaviours where this mental link is particularly strong may point to effective entry points for initially small and subsequently bigger behaviour changes. Take for example the pair of behaviours most endorsed in our study, compensating a full bath by taking shorter showers (NCGB1/ECGB1). We could imagine (as a thought experiment, precluding obvious data privacy issues) that whenever a water meter detects a full bath, this household could be contacted to consider their showering practices, as it is likely that they will heed this advice at this time. Subsequently, they could be approached with further advice on saving energy in other areas of hot water consumption. Thus, identifying pairs of behaviours with a strong compensatory link could indicate entry points for initiating cascading sequences of behavioural change. In order to identify these pairs, future studies could employ card sorting tasks (Gabe-Thomas et al., 2016; Seebauer & Ellmer, 2023), asking respondents to group various target behaviours with their respective compensatory behaviours.

### Limitations and Suggestions for Future Research

The present study comes with several methodological limitations. This study relies on self-reports of sustainable behaviour that might be coloured by the respondents’ attitudes. GCGBs were measured with just two moderately correlated items, which increased measurement error and enlarged confidence intervals of the respective coefficients. Moreover, the GCGB item wording presupposes that respondents lead an unsustainable lifestyle (which is fairly likely though considering the typical living situation of high school graduates in the Global North).

The original German phrasing of the NCGB items as “sollte” may be translated into English as “should” or “ought,” implying emotions of regret or guilt (Zhang et al., 2021). This nuance is not available in the German language. Thus, future studies using non-German samples could confirm whether the normative dimension of CGBs holds regardless of the specific emotion evoked.

NCGB responses might have primed ECGB responses because the same pairs of behaviours were matched between normative and efficacy items, and the normative and efficacy items were grouped in consecutive questionnaire blocks. Correlating the error terms of matched items need not fully control for this method effect; thus, the *r* = 0.39 correlation between NCGBs and ECGBs may be overestimated (Table 2). ECGB items may capture response efficacy, the ability of the behaviour to accomplish the desired outcome, as well as self-efficacy, the perceived ability of the respondent to perform the behaviour.

The temporal sequence between target and compensatory behaviour is ambiguous in some items. In NCGBs and ECGBs, the items 1 and 4 explicitly put the compensating behaviour as succeeding the target behaviour following Dolan & Galizzi’s (2015) logic of moral cleansing or purging, whereas in item 3, the compensating behaviour precedes the target behaviour, following the logic of moral licensing or permitting. However, the factor loadings of items 1, 3, and 4 do not stand out against the other items, suggesting they constitute a common factor nevertheless (Table 2). By contrast, all GCGB items are worded as morally licensing in that doing something for the environment precedes refraining from further efforts. This divergence in temporal sequence could explain the absent effect of GCGBs on sustainable behaviours (Table 4), because the permitting logic builds on concrete and tangible actions rather than abstract general compensatory beliefs (Dolan & Galizzi, 2015). Further studies should avoid ambiguity by operationalizing CGBs clearly as either morally cleansing or licensing.

The quiz questions on environmental knowledge seemed fairly easy (6 of 8 questions had a > 50% rate of correct answers; Table 7 in the Appendix). Providing only three answer options in each quiz question may have facilitated guessing the correct answer. Too easy quiz questions may have decreased variance in the knowledge score, possibly explaining the weak correlations of environmental knowledge with CGBs (Table 3) and the weak effects of environmental knowledge on sustainable behaviours (Table 4).

It should be kept in mind that the present study focuses on efficacy compensatory beliefs, not effective compensatory actions, or, on the mental accounting in balancing amends with missteps, not the real-world accounting how the carbon impacts of specific behaviours cancel each other out. According to their nature as beliefs, efficacy compensatory beliefs presume ideal and abstract circumstances under which the compensatory behaviour would be performed. For instance, the ECGB2 statement “I can reduce the CO2 consumption of my flights abroad by using only public transport at home” reflects a generalized, situation-independent mindset. By contrast, effective compensatory actions need to refer to the real-world conditions the respondent lives in. When operationalizing the ECGB2 statement as an effective compensatory action, respondents could be asked how many flights they undertook last year and whether public transport is a viable option on their daily routes, and the item could be adapted accordingly in a dynamic online questionnaire, possibly even displaying numerically the specific tons of CO_2_ emitted by flying and the number of kilometres by public transport necessary for offsetting. Future research could measure both compensatory beliefs and actions in order to address the extent of compensatory fallacy, that is, to what extent a compensatory behaviour is wrongly believed to actually offset the target behaviour.

## Data Availability

Data can be obtained from the authors upon reasonable request.

## Appendix

Please see Tables 6, 7, 8, 9, 10, 11 and 12.

**Table 6:** Sample descriptives

	Female		Age (years)						Education of parents			
		16	17	18	19	20	21	22	Comp	Voc	Sec	High
*N* = 502, one-wave t1 (cross-sectional data, all)	51.6	0.2	27.6	46.5	22.1	3.0	0.6	-	4.2	23.8	35.0	37.1
*N* = 319, one-wave t1 (cross-sectional data, completed in classroom)	43.5	0.3	28.8	41.8	24.9	3.5	0.6	-	4.6	24.1	35.3	36.0
*N* = 183, one-wave t1 (cross-sectional data, completed at home)	68.2	-	25.3	55.8	16.2	1.9	0.6	-	3.4	22.8	34.5	39.3
*N* = 145, first wave t1 (longitudinal data)	57.9	-	31.7	46.2	18.6	2.8	0.7	-	2.1	17.1	27.9	52.9
*N* = 145, second wave t2 (longitudinal data)	58.7	-	-	27.5	48.6	21.0	2.2	0.7				

The table gives relative frequencies in percent. Education of parents: highest educational level attained by either parent. *Comp.*, compulsory education; *Voc.*, vocational education; *Sec.*, secondary school (with school-leaving exam); *High*, higher education (university-level)

**Table 7:** Item wordings and descriptives

Construct	Item	*N* = 502, one-wave t1 (cross-sectional data)				*N* = 145, first wave t1 (longitudinal data)			*N* = 145, second wave t2 (longitudinal data)		
		*N*	Mean	SD	Loading	*N*	Mean	SD	*N*	Mean	SD
General compensatory green beliefs	As long as I take a few simple actions to protect the climate, that is enough. (GCGB1) §	478	2.57	1.00	0.79	-	-	-	-	-	-
	I already try to do my part in climate protection and I am not willing to adapt my lifestyle any further. (GCGB2)	474	2.44	1.00	0.30	-	-	-	-	-	-
Normative compensatory green beliefs	If I take a full bath every week, I should not shower too long on the remaining days. (NCGB1)	482	3.88	1.28	0.55	-	-	-	-	-	-
	When I fly abroad, I should only use public transport at home. (NCGB2)	482	3.08	1.30	0.81	145	3.31	1.20	145	3.35	1.09
	If I buy imported products at the supermarket, I should get there on foot or by bicycle. (NCGB3) §	482	2.93	1.33	0.80	145	3.21	1.26	145	3.14	1.21
	I should compensate for the purchase of new electrical devices by always disconnecting these devices from the mains when not using them. (NCGB4) #	482	3.55	1.27	0.65	145	3.78	1.20	143	3.54	1.23
	If I travel by air, I should pay a CO2 compensation. (NCGB5)	482	3.24	1.38	0.53	-	-	-	-	-	-
Efficacy compensatory green beliefs	I can reduce the energy consumption of a weekly full bath by taking a short shower on the remaining days (ECGB1)	476	3.62	1.17	0.70	-	-	-	-	-	-
	I can reduce the CO2 consumption of my flights abroad by using only public transport at home (ECGB2)	476	3.18	1.28	0.78	-	-	-	-	-	-
	I can reduce the climate impact of buying imported products by walking or cycling to the supermarket (ECGB3)	476	3.04	1.26	0.78	-	-	-	-	-	-
	I can compensate for the purchase of new electrical products by always disconnecting these devices from the mains when not using them (ECGB4)	476	3.22	1.29	0.68	-	-	-	-	-	-
	I can reduce the CO2 emissions of air travel by paying a CO2 compensation (ECGB5)	476	2.62	1.38	0.55	-	-	-	-	-	-
Environmental self-identity	If I had to describe myself, my mindset about climate protection would play a role. §	484	2.90	1.10	0.66	145	3.20	1.06	145	3.02	1.17
	I see it as my responsibility to contribute to climate protection	484	3.69	1.10	0.81	145	4.06	0.97	145	3.97	0.85
	I see myself as a person who considers climate protection. #	478	3.69	1.07	0.83	145	4.01	0.95	143	3.90	1.03
Social norms	People that are important to me contribute to climate protection. §	484	3.01	1.04	0.80	145	3.40	0.93	145	3.37	0.96
	People that are close to me make an effort to contribute to climate protection	474	3.02	1.08	0.87	145	3.34	1.00	141	3.40	0.96
	People that are important to me think I should contribute to climate protection. #	474	2.66	1.14	0.73	145	3.00	1.12	141	3.04	1.17
	People that are close to me appreciate it if I consider climate protection	478	3.42	1.07	0.67	145	3.70	0.92	143	3.73	0.88
Pro-environmental values	The balance of nature is strong enough to cope with the impacts of modern industrial nations. $ §	484	1.66	0.91	0.43	145	1.56	0.85	145	1.52	0.78
	Humans were meant to rule over the rest of nature. $	478	1.91	1.11	0.32	145	1.85	1.09	-	-	-
	If we continue our current style of living, we are approaching an environmental catastrophe	474	4.16	1.05	0.72	145	4.37	0.91	141	4.52	0.80
Environmental knowledge	Which household domain consumes the most energy? a) **heating b)** lighting c) water heating)	473	0.55	0.49	-	-	-	-	-	-	-
	What consumes the least energy when heating water? a) **Electric kettle** b) Electric stove c) Gas stove	473	0.53	0.49	-	-	-	-	-	-	-
	How many percent of reduction in CO2 emission can be achieved through a plastic bag ban? a) 1% b) 4% c) **0.01%**	473	0.18	0.38	-	-	-	-	-	-	-
	Which of the following materials is best suited for recycling? a) **Aluminium** b) Plastic bottles c) Green glass	473	0.15	0.36	-	-	-	-	-	-	-
	What is the most energy-efficient way to ventilate in winter? a) **Leave windows open for up to 10 min** b) Leave the window open for more than 10 min c) Leave windows tilted for more than 10 min	473	0.86	0.34	-	-	-	-	-	-	-
	The carbon emissions of a 100 km car journey correspond to a) 12 km train ride b) 120 km train ride c) **1.200 km train ride**	473	0.63	0.48	-	-	-	-	-	-	-
	How much lower is the carbon footprint of organic milk compared to conventional milk? a) 10% b) **30%** c) 70%	473	0.66	0.47	-	-	-	-	-	-	-
	Which type of meat has the largest CO_2_ footprint? a) **Beef** b) Pork c) Chicken	473	0.75	0.43	-	-	-	-	-	-	-
Food consumption	Eat meat products $ §	502	3.42	1.25	0.51	145	3.11	1.31	145	2.91	1.23
	Buy organic food products #	501	3.45	1.07	0.65	145	3.72	0.86	145	3.70	0.83
	Buy products in reusable packaging such as returnable bottles	502	3.42	0.83	0.53	145	3.55	0.73	145	3.43	0.79
	Avoid plastic packaging	501	3.23	0.86	0.62	145	3.29	0.82	145	3.30	0.76
Indulging behaviours	Have my parents fetch me by car after a night out §	502	2.30	1.26	0.30	145	2.15	1.19	145	1.90	0.97
	Buy new clothes and shoes or get them as a present to be always dressed fashionably	502	2.94	1.05	0.80	145	2.66	0.92	145	2.52	0.96
	Buy new electronic devices or get them as a present to always have the best available technology #	501	2.41	1.04	0.57	145	2.24	0.99	145	2.29	1.00
Waste separation behaviours	Not throw anything in the residual waste for which there are separate bins §	502	4.01	1.12	0.45	145	4.17	1.08	145	4.28	0.80
	Throw paper only in the paper waste bin #	501	4.33	0.95	0.59	145	4.50	0.82	145	4.50	0.81
Repair and reuse behaviours	Have broken electronic devices repaired or repair them myself instead of discarding them §	502	3.33	1.14	0.49	145	3.45	1.14	145	3.50	1.01
	Have damaged clothes and shoes repaired or repair them myself instead of discarding them #	501	2.65	1.17	0.75	145	2.86	1.17	145	3.08	1.16
	Buy used electronic devices	501	2.04	1.01	0.32	145	2.06	0.97	145	2.26	1.09
Energy use behaviours	Turn off the lights when not needed §	502	4.49	0.75	0.43	145	4.50	0.71	145	4.45	0.67
	Turn off the hot water while soaping under the shower #	502	3.33	1.66	0.43	145	3.39	1.63	145	3.54	1.55
	Disconnect electronic devices from the mains when not using them	501	2.97	1.30	0.55	145	3.12	1.24	145	3.20	1.14
	Take shorter showers to save hot water	501	2.81	1.20	0.55	145	3.00	1.11	145	3.01	1.09

The table gives standardized factor loadings. Five-step response scale with endpoints labelled 1 = fully disagree and 5 = fully agree; behaviours with endpoints 1 = never and 5 = always; knowledge with 0 = wrong and 1 = correct. *N* = valid responses. *SD*, standard deviation. $ Item reverse-coded in the analyses due to negative wording. § Marker item with factor loading fixed to 1. # Time-invariant factor loading, constrained to be equal at t1 and t2 to establish partial measurement invariance over time (Byrne et al., 1989; Steenkamp & Baumgartner, 1998). Correct answers in quiz questions printed bold. Factor loadings refer to the food consumption model and vary in the other models only by the second decimal place

**Table 8:** Power analysis

Scenario	Assumptions	Results
Confirmatory factor analysis of CGBs and other constructs (corresponds to Table 3)	Intercorrelations of other constructs: *r* = 0.30 Factor loadings 0.70, reflecting the observed magnitudes (see Table 7) Error covariances 0.00, because the method effect of similarly phrased paired NCGB-ECGB items would not be present in the population 1000 simulations	
Intercorrelations of NCGB, ECGB, and GCGB of *r* = 0.30		Power of > 0.99 for NCGB, ECGB, and GCGB
Structural equation model explaining food consumption from CGBs and other constructs (corresponds to Table 4), because this behaviour is measured with the maximum number of indicators Effect of NCGB, ECGB, and GCGB on sustainable behaviour of	As above, plus: Other structural paths: β = 0.20	
β = 0.10		Power of 0.35 for NCGB, 0.31 for ECGB, 0.26 for GCGB
β = 0.15		Power of 0.60 for NCGB, 0.61 for ECGB, 0.46 for GCGB
β = 0.20		Power of 0.82 for NCGB, 0.85 for ECGB, 0.73 for GCGB

Power calculated using the pwrSEM application (Wang & Rhemtulla, 2021)

**Table 9:** Principal component analysis of CGB items

Item	Factor 1	Factor 2	Factor 3
If I take a full bath every week, I should not shower too long on the remaining days (NCGB1)		0.77	
When I fly abroad, I should only use public transport within the country (NCGB2)		0.77	
If I buy imported products at the supermarket, I should get there on foot or by bicycle (NCGB3)		0.80	
I should compensate for the purchase of new electrical products by always disconnecting these devices from the mains when I am not using them (NCGB4)		0.74	
If I fly, I should pay a CO2 compensation (NCGB5)		0.56	− 0.30
I can reduce the energy consumption of a weekly full bath by taking a short shower on the remaining days (ECGB1)	0.72		
I can reduce the CO2 consumption of my international flights by travelling only by public transport at home (ECGB2)	0.82		
I can reduce the climate impact of buying imported products by walking or cycling to the supermarket (ECGB3)	0.83		
I can reduce the purchase of new electrical products by always disconnecting these devices from the mains when I am not using them (ECGB4)	0.76		
I can reduce the CO2 emissions of a flight by paying a CO2 compensation (ECGB5)	0.66		
As long as I take a few simple actions to protect the climate, that is enough. (GCGB1)			0.74
I already try to do my part in climate protection and I am not willing to adapt my lifestyle any further. (GCGB2)			0.73
Variance explained (%)	0.25	0.23	0.11
Eigenvalue	3.93	2.06	1.01

Oblimin rotation. Factor loadings < 0.30 omitted

**Table 10:** Robustness check: confirmatory factor analysis of normative, efficacy, and general CGBs with single-item measure of GCGBs

	Coefficient	95% CI	AVE	CR
Normative beliefs			0.46	0.80
NCGB1	0.55***	[0.48, 0.62]		
NCGB2	0.81***	[0.76, 0.86]		
NCGB3	0.80***	[0.75, 0.84]		
NCGB4	0.65***	[0.59, 0.72]		
NCGB5	0.53***	[0.44, 0.61]		
Efficacy beliefs			0.49	0.82
ECGB1	0.70***	[0.63, 0.76]		
ECGB2	0.78***	[0.72, 0.84]		
ECGB3	0.78***	[0.72, 0.84]		
ECGB4	0.68***	[0.61, 0.75]		
ECGB5	0.55***	[0.47, 0.62]		
General beliefs				
GCGB1^a^	0.90***	[0.80, 0.91]		
Latent variable correlations				
NCGB-ECGB	0.39***	[0.27, 0.50]		
NCGB-GCGB	− 0.23**	[− 0.35, − 0.11]		
ECGB-GCGB	0.11	[− 0.01, 0.23]		

Table 10 gives standardized factor loadings and latent variable correlations. *N* = 482, one-wave cross-sectional data. ****p* < 0.001; ***p* < 0.01. 95% confidence interval in square brackets. χ^2^ = 295.46, df = 185; RMSEA = 0.036 [90% CI: 0.028; 0.042]; SRMR = 0.045; CFI = 0.968. *AVE*, average variance extracted. *CR*, composite reliability. Table 10 refers to the same confirmatory factor analysis as in Table 11. ^a^Single-item error variance fixed to 20% (Andrews, 1984)

**Table 11:** Robustness check: confirmatory factor analysis of normative, efficacy, and general CGBs with environmental knowledge, environmental self-identity, social norms, and pro-environmental values

	NCGB	ECGB	GCGB^a^
Environmental knowledge	0.03 [− 0.07, 0.12]	− 0.12* [− 0.22, − 0.02]	− 0.13* [− 0.22, − 0.03]
Environmental self-identity	0.49*** [0.40, 0.59]	0.16* [0.03, 0.28]	− 0.37*** [− 0.48, − 0.25]
Social norms	0.32*** [0.21, 0.42]	0.11 [− 0.01, 0.23]	− 0.33*** [− 0.43, − 0.22]
Pro-environmental values	0.35*** [0.22, 0.49]	0.09 [− 0.05, 0.24]	− 0.33*** [− 0.47, − 0.18]

Table 11 gives latent variable correlations. *N* = 482, one-wave cross-sectional data. ****p* < 0.001; ***p* < 0.01; **p* < 0.05. 95% confidence interval in square brackets. χ^2^ = 295.46, df = 185; RMSEA = 0.036 [90% CI: 0.028; 0.042]; SRMR = 0.045; CFI = 0.968. Table 11 refers to the same confirmatory factor analysis as in Table 10. ^a^GCGB measured with the single-item GCGB1 with error variance fixed to 20%

**Table 12:** Robustness check: structural equation modelling explaining sustainable behaviours from normative, efficacy, and general CGBs, and other constructs

	Food consumption	Indulging	Waste separation	Repair and reuse	Energy use	General behaviour
Environmental knowledge	− 0.00 [− 0.10, 0.08]	− 0.16** [− 0.27, − 0.06]	− 0.05 [− 0.18, 0.08]	0.03 [− 0.08, 0.14]	− 0.13 [− 0.24, − 0.02]	− 0.11** [− 0.18,. − 03]
Environmental self-identity	0.51*** [0.28, 0.74]	0.02 [− 0.25, 0.29]	0.57** [0.19, 0.95]	0.14 [− 0.12, 0.40]	0.50*** [0.21, 0.79]	0.47*** [0.28, 0.67]
Social norms	0.29*** [0.15, 0.43]	0.04 [− 0.13, 0.21]	− 0.20 [− 0.43, 0.03]	0.09 [− 0.09, 0.27]	− 0.06 [− 0.24, 0.11]	0.06 [− 0.07, 0.19]
Pro-environmental values	0.05 [− 0.16, 0.26]	− 0.09 [− 0.35, 0.18]	− 0.05 [− 0.34, 0.24]	− 0.00 [− 0.25, 0.22]	− 0.16 [− 0.44, 0.12]	− 0.08 [− 0.25, 0.10]
NCGB	0.14* [0.00, 0.28]	− 0.28** [− 0.48, − 0.08]	0.34** [0.08, 0.59]	0.23** [0.06, 0.40]	0.36*** [0.18, 0.54]	0.22*** [0.09, 0.34]
ECGB	− 0.07 [− 0.18, 0.04]	0.24** [0.11, 0.37]	− 0.08 [− 0.25, 0.08]	− 0.08 [− 0.23, 0.07]	0.02 [− 0.12, 0.17]	0.00 [− 0.09, 0.34]
GCGB ^a^	− 0.01 [− 0.14, 0.11]	0.04 [− 0.10, 0.18]	0.11 [− 0.06, 0.29]	− 0.15* [− 0.29, − 0.01]	− 0.00 [− 0.15, 0.15]	− 0.02 [− 0.12, 0.08]
*R*^2^ (*R*^2^ without CGB)	0.69 (0.67)	0.15 (0.04)	0.39 (0.27)	0.19 (0.12)	0.43 (0.32)	0.38 (0.35)
χ^2^ (df)	453.6 (268)	406.1 (244)	352.2 (221)	364.3 (244)	450.2 (268)	335.3 (200)
RMSEA [90% CI]	0.039 [0.032, 0.045]	0.038 [0.031, 0.045]	0.036 [0.029, 0.043]	0.033 [0.025, 0.040]	0.039 [0.032, 0.045]	0.039 [0.031, 0.046]
SRMR	0.048	0.050	0.044	0.044	0.046	0.045
CFI	0.945	0.956	0.964	0.967	0.951	0.961

Table 12 gives standardized path coefficients. *N* = 502, one-wave cross-sectional data. ****p* < 0.001; ***p* < 0.01; **p* < 0.05. 95% confidence interval in square brackets. ^a^GCGB measured with the single-item GCGB1 with error variance fixed to 20%
